# Genomic and proteomic evidence supporting the division of the plant pathogen *Ralstonia solanacearum* into three species

**DOI:** 10.1186/s12864-016-2413-z

**Published:** 2016-02-01

**Authors:** Philippe Prior, Florent Ailloud, Beth L. Dalsing, Benoit Remenant, Borja Sanchez, Caitilyn Allen

**Affiliations:** UMR PVBMT Peuplements Végétaux et Bioagresseurs en Milieu Tropical, CIRAD, Saint Pierre, La Réunion France; Anses–Plant Health Laboratory (LSV), 7 chemin de l’IRAT, Saint-Pierre, La Réunion France; Department of Plant Pathology, University of Wisconsin-Madison, 1630 Linden Drive, Madison, WI 53706 USA; Department of Plant Health and Environment (SPE), INRA, Paris, France; Department of Analytical and Food Chemistry, University of Vigo, Ourense, Spain

**Keywords:** *Ralstonia solanacearum*, Bacterial wilt, Plant pathogen, Taxonomy, Genomics, Proteomics

## Abstract

**Background:**

The increased availability of genome sequences has advanced the development of genomic distance methods to describe bacterial diversity. Results of these fast-evolving methods are highly correlated with those of the historically standard DNA-DNA hybridization technique. However, these genomic-based methods can be done more rapidly and less expensively and are less prone to technical and human error. They are thus a technically accessible replacement for species delineation. Here, we use several genomic comparison methods, supported by our own proteomic analyses and metabolic characterization as well as previously published DNA-DNA hybridization analyses, to differentiate members of the *Ralstonia solanacearum* species complex into three species. This pathogen group consists of diverse and widespread strains that cause bacterial wilt disease on many different plants.

**Results:**

We used three different methods to compare the complete genomes of 29 strains from the *R. solanacearum* species complex. In parallel we profiled the proteomes of 73 strains using Matrix-Assisted Laser Desorption/Ionization-Time of Flight Mass Spectrometry (MALDI-TOF-MS). Proteomic profiles together with genomic sequence comparisons consistently and comprehensively described the diversity of the *R. solanacearum* species complex. In addition, genome-driven functional phenotypic assays excitingly supported an old hypothesis (Hayward et al. (J Appl Bacteriol 69:269–80, 1990)), that closely related members of the *R. solanacearum* could be identified through a simple assay of anaerobic nitrate metabolism. This assay allowed us to clearly and easily differentiate phylotype II and IV strains from phylotype I and III strains. Further, genomic dissection of the pathway distinguished between proposed subspecies within the current phylotype IV. The assay revealed large scale differences in energy production within the *R. solanacearum* species complex, indicating coarse evolutionary distance and further supporting a repartitioning of this group into separate species.

**Conclusions:**

Together, the results of these studies support the proposed division of the *R. solanacearum* species complex into three species, consistent with recent literature, and demonstrate the utility of proteomic and genomic approaches to delineate bacterial species.

**Electronic supplementary material:**

The online version of this article (doi:10.1186/s12864-016-2413-z) contains supplementary material, which is available to authorized users.

## Background

Thousands of genetically distinct strains within the *Ralstonia solanacearum* species complex (RSSC) cause bacterial wilt diseases in plants. These bacteria colonize the xylem tissue of host plant vascular systems causing stunting, wilting, yield reduction, and death. This pathogen group has major economic and social impact worldwide [2, 3]. Members of the RSSC can collectively infect over 250 hosts in 54 botanical families and include: *R. solanacearum* strains, which collectively infect a broad host range and are typically soil-borne; *R. syzygii*, a spittlebug-transmitted pathogen that causes Sumatra disease in cloves; and the Blood Disease Bacterium (BDB), an unclassified organism responsible for the pollinator-transmitted Blood Disease of bananas and plantains in the Philippines.

Smith first described the morphological and chemotaxonomic characteristics of the bacterial wilt pathogen as *Bacterium solanacearum*, and this species has most recently been placed in the genus *Ralstonia* [4, 5]*.* The BDB was described and named *Pseudomonas celebensis* in 1921 [6, 7]. However this name lost its standing in nomenclature when the original strain was lost so there was no authentic type strain. The Sumatra disease pathogen, originally described as *Pseudomonas syzygii*, was placed in a separate species in the genus *Ralstonia* based on 16S sequences and DNA-DNA hybridization (DDH) data showing substantial divergence from *R. solanacearum* [8]. However, the DDH study that concluded *R. syzygii* should be placed in a separate species was based on a comparison with *R. solanacearum* K60^T^, a phylotype II strain that is quite phenotypically and genotypically divergent from *R. syzygii*, a member of phylotype IV.

DNA-DNA hybridization has been used to distinguish species since the 1960s, contributing importantly to the modern bacterial species concept [7, 9]. However, because complete sequenced genomes contain significantly more information than can be inferred from the results of DDH and computer-driven methods are less prone to human error, this technique can now be replaced with bioinformatics methods [10, 11]. Early analyses based on the single-gene phylogeny of the conserved *egl, mutS, hrpB* or ITS sequences divided the RSSC into four distinct genospecies, known as phylotypes, corresponding to strain geographic origin: phylotype I (Asia), phylotype II (Americas), phylotype III (Africa), and phylotype IV (Indonesia and Japan) [12–15]. The phylogenetic structure of the RSSC was subsequently confirmed in an extensive series of genomic studies involving a large array of analytical methods from microsatellites and MLST to microarrays [13, 16, 17].

While the analyses of the complete genome sequences of several strains in the RSSC provides strong evidence supporting the phylotype structure [9, 12, 18–20], they further reveal a larger degree of evolutionary divergence among the phylotypes that warrants the division of the RSSC into three species, as previously suggested [19]. Recently, Safni et al., [21] supported this taxonomic revision, calling for an amendment of the descriptions of the RSSC based on a polyphasic approach with emphasis on DNA-DNA hybridization analysis. Safni suggested that *R. pseudosolanacearum* sp. nov., corresponding to phylotypes I and III, and *Ralstonia syzygii*, corresponding to phylotype IV should be considered two species. Based on differences in pathological phenotype, they suggested that *R. syzygii* be further divided into three subspecies. The broad host-range soil-borne strains were proposed to be renamed *R. syzygii* subspecies *indonesiensis* subsp. nov. The unclassified banana Blood Disease Bacterium was proposed to be named *R. syzygii* subspecies *celebesensis* subsp. nov. and *R. syzygii*, which causes Sumatra disease in cloves, was proposed to be renamed *R. syzygii* subspecies *syzygii* subsp. nov. Finally, Safni called for phylotype II strains (from the Americas), which include the species type strain K60^T^ (=ATCC11696^T^ = LMG2299^T^), to remain in *R. solanacearum.*

In the present study, we used a combination of genomic and proteomic methods and a large genome pool to unambiguously delineate species within the RSSC. We formally examined these methods for their correspondence to the recent reclassification of this taxonomically disputed organism into three distinct species based on DNA-DNA hybridization. This work validates the re-distribution of *R. solanacearum* into three species based on modern methods. We further provide tools for the rapid identification and classification of new isolates into species and subspecies without DNA-DNA hybridization.

## Results and discussion

### Phenotypic diversity in the RSSC

Safni et al., [21] analyzed the RSSC using phenotype microarrays and identified major variation in the core metabolisms both between and within phylotypes, which generally supported the idea that *R. solanacearum* can be divided into multiple species but no assay was able to provide clear distinction between the three proposed species. In the present study, we carefully dissected functional as well as genotypic differences in the denitrification metabolic pathway. This pathway is associated with several quantifiable and biologically relevant phenotypic traits that play major roles in virulence of a phylotype I strain [22]. Additionally, these phenotypes were known to vary among strains prior to modern-day genome sequencing and phylotyping [1].

In 1990, Hayward recognized variability in anaerobic nitrogen metabolism between *R. solanacearum* strains [23]. However, the biovar sub-classification system, in use at the time, did not correspond to the phylogenetic relationships among RSSC strains. No clear patterns differentiated biovars by anaerobic nitrogen metabolism, thus denitrification was not considered a useful trait for strain typing. Recent phylogenetic analyses that reclassified strains into phylotypes, and here into species, motivated us to revisit the idea that this metabolic trait could be used to differentiate phylogenetically distinct groups.

**Fig. 1:**
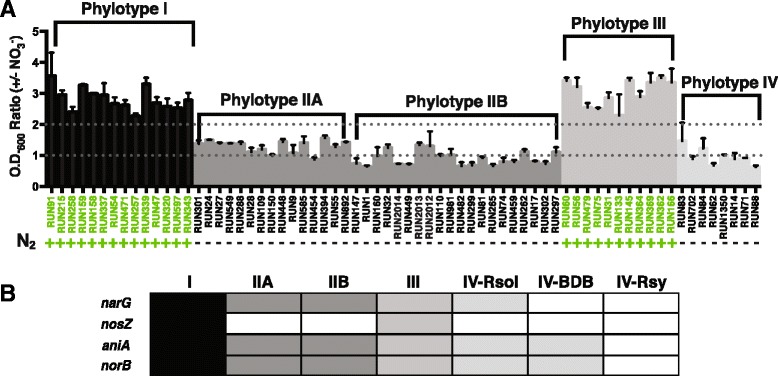
The denitrification phenotype across the RSSC. **a** Growth and production of nitrogen gas under anaerobic condition. Values represent the ratio of O.D._600_ readings following 72 h of anaerobic incubation in VDM plus 30 mM NO_3_^−^ vs. without added NO_3_^−^. A value above 1 indicates that in the presence of NO_3_^−^ a strain reached higher optical densities than in the absence of NO_3_^−^, indicating NO_3_^−^ respiration enhanced growth. A value above the arbitrary threshold of 2 meets our cut-off for biological significance. Strain names in *green* and *green* ‘+’ *s* indicate N_2_ gas was produced within 96 h of anaerobic inoculation in VDM + 30 mM NO_3_^−^. Production of N_2_ indicates that the strain completed the full denitrification pathway as indicated by production of visible N_2_ gas bubbles. *Bars* indicate standard error. Data represent the means of 4–6 biological replicates. **b** Summary of the presence/absence of denitrification genes. *Black/gray cells* indicate the presence of a gene in all the sequenced strains of this group and white cells indicate its absence

Denitrification is an anaerobic respiration process that allows strains to use nitrate as a terminal electron acceptor to grow under anaerobic conditions. Nitrate (NO_3_^−^) is converted successively to nitrite (NO_2_^−^), nitric oxide (NO^.^), nitrous oxide (N_2_O), and finally nitrogen gas (N_2_) in a series of four reactions catalyzed by the products of the *narG*, *aniA*, *norB* and *nosZ* genes, respectively. To determine which strains were able to use denitrification for energy and growth, we incubated 68 strains (14 phylotype I; 35 phylotype II; 11 phylotype III; 8 phylotype IV) anaerobically both with and without nitrate. After 72 h, we measured O.D._600_ and calculated the ratio of endpoint optical density in cultures provided with nitrate compared with those lacking a traditional terminal electron acceptor. Strains with a ratio equal to or above two were considered able to respire on nitrate under these conditions. All tested strains in phylotypes I and III respired on nitrate but no strains within phylotypes II and IV exhibited this trait (Fig. 1a).

Our phenotypic data on nitrogen metabolism in the RSSC conflict with those presented by Safni et al. Their formal proposed new and emended species descriptions state that “some” strains of phylotype II and “most” strains of the proposed *R. syzygii* subsp. indonesiensis in phylotype IV can reduce nitrate to nitrogen gas, while “most” strains of phylotypes I and III (proposed *R. pseudosolanacearum*) can reduce nitrate to nitrogen gas. In contrast, our biochemical assays of 68 diverse RSSC strains consistently found that no strains in phylotypes II and IV could complete denitrification under anaerobic conditions, while all strains in phylotypes I and III can do so. These contrasting results may reflect differences between our denitrification assays [22] and the 1964 Hayward method used by Safni et al.

The denitrification phenotypes were consistent with genomic analysis showing that all sequenced phylotype I and III strains have a gene (*narG)* encoding the major catalytic subunit of a respiratory nitrate reductase (Fig. 1b). Sequenced phylotype II strains do harbor *narG*, but the functional analysis described above, indicates that these strains either do not make a functional enzyme or do not use this nitrate reductase under the anaerobic conditions tested here. Phylotype IV strains are genomically divided into three groups based on presence or absence of the nitrate (*narG*), nitrite (*aniA*), and nitric oxide (*norB*) reductase genes that encode the first three steps of the pathway. All sequenced strains of the proposed *R. syzygii* subspecies *indonesiensis* (currently phylotype IV *R. solanacearum*) contain *narG*, but strains of the proposed *R. syzygii* subspecies *celebesensis* (currently phylotype IV BDB) lack this gene. Strains of the proposed *R. syzygii* subspecies *syzygii* (currently phylotype IV *R. syzygii*) lack *narG*, *aniA*, and *norB*.

We used functional analyses to determine if each strain could denitrify completely. Complete denitrification, the full step-wise conversion of nitrate to dinitrogen, is indicated by the production of visible dinitrogen gas bubbles. Importantly, this gas was absent in all cultures that were not provided nitrate. Phylotypes I and III did complete denitrification, but no members of phylotypes II and IV did (Fig. 1a). This finding correlated perfectly with our genomic observation that all sequenced phylotype I and III strains contain *nosZ,* encoding a nitrous oxide reductase while no sequenced phylotype II or IV strains harbor *nosZ* (Fig. 1b).

From an evolutionary perspective, it appears that these three proposed species have adapted to use different energy production mechanisms. Phylotypes I and III use denitrification under anaerobic conditions for energy while phylotypes II and IV do not. Furthermore, phylotype II strains have maintained genes that suggest they regulate this metabolism differently. Phylotype IV strains appear to be in the process of losing this pathway altogether. This step-wise loss of denitrification may be associated with the ability to be vector transmitted, like *R. syzygii* subsp. *syzygii*. Alternatively, if the pathway is being gained step-by-step, denitrification may enhance soil survival and transmission via root entry. These hypotheses are being further explored. As Hayward proposed in 1990, groups in the RSSC can be distinguished all the way down to the subspecies level by a combination of functional and genomic evaluation of a single pathway, denitrification.

### Genomic diversity in the RSSC

The 16S rRNA gene sequences of *R. solanacearum* strains are more than 97 % identical, suggesting that this group forms a single species that is distinct from its close relative *R. eutropha* [5]. However, 16S rRNA sequences do not always accurately reflect similarities at the whole-genome level and they cannot distinguish between recently diverged species [24, 25]. Moreover, this identity threshold has not been universally accepted, and distinct species with 98 % identical 16S rRNA sequences have been described [23]. DDH was historically used for species delineation, and a 70 % DNA-DNA similarity was traditionally used to define species. Recently, Safni et al. argued that *R. solanacearum* can be divided into 3 species based on DDH values. In a complementary approach, we evaluated the taxonomy of *R. solanacearum* using genomic and proteomic data. As a method, DDH has significant drawbacks: it is technically difficult, is performed only in a few specialized laboratories, and is prone to experimental errors [26]. DDH assays can only measure the potential for hybridization between purified DNA from two organisms, without regard to biological function. Thus, the 70 % DDH criterion does not correspond to 70 % shared orthologous genes or even 70 % sequence identity [27]. Strains showing more than 70 % DDH can possess up to 21 % divergent gene content, which is equivalent to around 1000 genes in a typical 5.3 Mb *R. solanacearum* genome [28]. With the recent development of *in silico* comparative methods using complete genome sequences, DDH is no longer the most reliable method for determining relatedness between bacterial strains.

In a previous study [19], we proposed division of the RSSC into 3 genomospecies based on a genome-to-genome comparison using Average Nucleotide Identity (ANI) analyses of 8 strains. In the present study, we included 15 additional genomes in the ANI analysis and compared the ANI data to two other genomic analysis methods showing a better correlation with DDH: the Maximum Unique Matches index (MUMi) and the Genome-to-Genome Distance Calculator (GGDC) [11, 29–31] (Additional file 1).

Briefly, ANI detects the level of conservation or similarity of the total genomic sequences shared between two strains based on the identification of homologous fragments of fixed length using the BLAST algorithm. Strains with ANI >95 % are considered as belonging to the same species, consistent with the 70 % DDH criterion [11, 28, 30, 32–34]. Like DDH, ANI accounts for the variability in conserved gene content but does not always reflect differences between closely related strains and strains with similar ANI values can have similar or dissimilar gene content [35]. The MUMi algorithm overcomes this problem by accounting for both the variability of homologous gene content and the gain and loss of DNA. MUMi distances are derived from a list of maximum unique matches (MUMs) of a given minimal length shared between two genomes and the average length between genomes. Because this technique uses a fast algorithm to detect MUMs, MUMi is significantly faster than ANI. A MUMi value of 0.33 ± 0.03 corresponds to an ANI value of 95 %. Finally, the recently revised GGDC method shows the highest correlation with wet-lab DDH [31, 36]. The GGDC also infers *in silico* DDH values from genomic distances; therefore, a similar 70 % threshold can be used. Although this method is based on principles similar to ANI and MUMi, GGDC uses a different set of formulas to estimate genomic distances.

The ANI values obtained from pairwise comparisons between all genomes are presented in Additional file 1. The 29 strains analyzed in the present study fall into three distinct groups. The first group includes strains from phylotypes I and III. The second group comprises phylotype II strains, divided into subgroups IIA (containing the current *R. solanacearum* type strain, K60^T^) and IIB. The last group includes phylotype IV strains (PSI07, BDB R229 and *R. syzygii* R24), originally described as a separate species before the establishment of the species complex. These ANI results are wholly consistent with a previous analysis of a smaller group of genomes [19].

The genomic distances calculated using the MUMi algorithm are presented in Additional file 1. This method separates the RSSC into 3 or more species depending on how strictly the 0.33 ± 0.03 criterion is applied. Consistent with the ANI analysis, this method identifies two distinct species: one containing phylotype IV, and one containing phylotypes I and III. However, the delineation of phylotype II as a single species was not definitive. The genomic distances were <0.33 ± 0.03 in 100 % of the strains within subgroups IIA and IIB but some genomic distances were >0.33 ± 0.03 between few strains of the two subgroups. Notably, the distances between IIB strain UW551 and 5 of the 6 IIA strains were above the threshold (0.37–0.40). However, we believe this reflects the relatively low quality of the UW551 genome, and not true biological or genomic diversity.

**Fig. 2:**
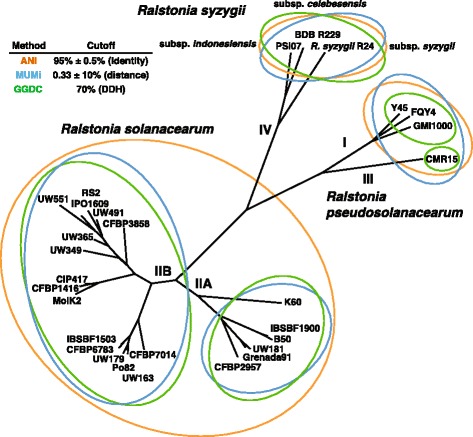
Phylogenetic network derived from genomic distances. The distance matrices were generated with ANI, MUMi and GGDC methods and the combined tree was created with the SplitsTree4 software. The *orange*, *blue* and *green* cells represent strains clustered into species using the criteria specific to the ANI, MUMi and GGDC methods, respectively

*In silico* DDH values inferred using the GGDC algorithm are presented in Additional file 1. According to the traditional 70 % DDH criterion, GGDC distinguishes 5 species within RSSC with phylotypes I, III and IV assigned to single species and phylotype II divided into 2 species corresponding to the IIA and IIB subgroups. Thus, GGDC more clearly differentiates between closely related strains. Although GGDC divides the RSSC into more species than ANI and MUMi, the raw result patterns are consistent between all 3 methods. As previously observed with the MUMi distances, IIA and IIB strains are definitely divergent at the whole-genome level. The ANI values between phylotypes I and III, or subtypes IIA and IIB, were approximately 96 %, while the ANI values within the species predicted using GGDC ranged from 97 to 99 %. To resolve these slightly varying analyses, we used SplitsTree software to build a phylogenetic network derived from the ANI, MUMi and GGDC distance matrices. The results showed no obvious ambiguities, confirming that all three genome sequence-based methods give broadly consistent results (Fig. 2).

The ANI, MUMi and GGDC methods are all based on whole-genome comparisons and have been shown to correlate well with the traditional standard method, DDH. Nonetheless, the RSSC can be divided into three to five species depending on how the genomic distances are calculated and the criteria used. Taken together, the outputs of these techniques illustrate the difficulty of consistently delineating species among closely related strains. Based on phenotypic data, we conclude that ANI and MUMi distances adequately reflect the level of biological variability within the RSSC, with a three-species division in which phylotypes I and III cluster together, and phylotypes II and IV are further apart.

### Proteomic diversity in the RSSC

A total of 73 bacterial strains representing the four phylotypes were subjected to comparative proteomic analysis as a complementary method to the genome-based analyses presented above (Additional file 2). Protein mass spectra corresponding to each strain were obtained using MALDI-TOF and clustered using SPECLUST software [37]. This generated a list of common peaks represented as inter-sample consensus m/z values. The best results were achieved using a “within peak match score (σ)” of 3 Da, as defined in the SPECLUST documentation. The consensus spectra matrix was translated to a binary matrix in which the absence/presence of a consensus peak in all strain profiles was represented as 0 or 1, respectively. This binary matrix was used to infer the phylogenetic relationships among the strains with the MALDI-TOF data and Bayesian analysis using MrBayes v3.2.2 software [38].

The MALDI-TOF approach was previously used for bacterial identification [39]. Mass fingerprinting is a simple, quick and reproducible method for bacterial identification through the generation of large spectral databases [40].

**Fig. 3:**
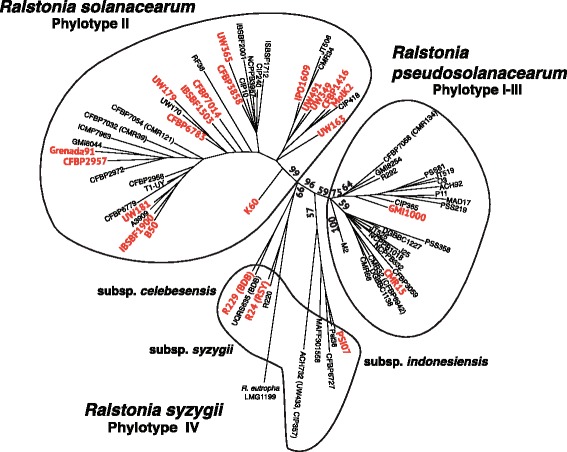
Tree derived from mass spectrometry analysis. Majority-rule consensus tree based on the presence/absence of a consensus MALDI-TOF peak list obtained using the MrBayes software. The probability values are indicated along the main branches. *Red* colored strains indicate the sequenced strains. *Black lines* delineate strain clusters

Taxonomically, molecular typing using protein profiles has been useful for bacterial classification at the species and subspecies levels [41, 42] and at the strain level, depending on the type and class of bacterial group considered [43]. In the present study, a combination of MALDI-TOF profiling, consensus mass peak lists, and Bayesian inference was used to cluster the 73 *Ralstonia* strains into three groups with strong branch support. The first cluster contained phylotypes I and III, whereas the second cluster contained phylotypes IIA and IIB, and the third cluster contained phylotype IV (Fig. 3). These results were consistent with the findings of the genomic analysis, supporting the division of *R. solanacearum* into three species.

### Phenotypic, genomic and proteomic data converge on a three-species model

The taxonomic classification of *R. solanacearum* has changed repeatedly over the last 50 years, grouping strains with divergent ecological, geographical, genetic and phenotypic profiles, including many pathological variants. Safni et al. recently used DDH for a taxonomic revision of the RSSC, proposing the division of this species complex into three distinct species. We used modern genomic techniques to explicitly reveal the phylogenetic relationships between strains and support a revision of the current taxonomy of this species complex. Moreover, phenotypic data such as measures of inorganic nitrogen metabolism can be directly correlated with genomic content in order to better understand the traits used to delineate species. Using a combination of phenotypic analyses, whole-genome comparisons and proteomic profiling, we provide additional information on the relationships between RSSC strains and offer useful phenotypic tests that distinguish among groups. The first species includes phylotypes I and III. The strains from these two phylotypes undergo denitrification, among other unique phenotypic properties, and are genetically closely related.

The second species corresponds to phylotype II. The strains in this phylotype are somewhat genomically divergent, resulting in the vague delineation of species based on genomic distances. Nonetheless, every strain belonging to phylotype II evaluated to date exhibits similar phenotypic properties and could therefore be considered a single species. The third species comprises: the phylotype IV strains currently classified as *R. solanacearum*; *R. syzygii,* which is transmitted through tube-building *Hindola* spp. cercopoid insects, with a host range limited to clove trees (Sumatra disease); and BDB, the causative agent of banana wilt diseases in Indonesia. Despite their strikingly different biological lifestyles [44], these strains are genetically related and share core metabolic activities. *R. solanacearum*, *R. syzygii* and BDB have different geographical distributions and pathogenic potential. Moreover, because these groups are easily genetically distinguishable, even when only considering one metabolic pathway-denitrification, the members of this third group could be considered subspecies.

## Conclusions

Extensive biological, phenotypic, and genetic data demonstrate that the RSSC is too diverse to be considered a single species. The modification of the taxonomy of this organism is necessary to recognize three phylogenetically distinct groups with different biological properties and evolutionary relationships. Newly isolated bacterial wilt strains can readily be assigned to the proposed scheme using existing molecular methods [14]. These changes will benefit many different applications, including breeding plant resistance to bacterial wilt, the identification of new pathological variants, management of quarantine containment and the development of diagnostic tests.

## Methods

The sequenced strains used in the present study are listed in Table 1. The strains used for the proteomic analyses are listed in Additional file 2. The phylotype placement of all strains was confirmed using the multiplex PCR method [14].

**Table 1:** *Ralstonia* spp. strains used in whole-genome analyses

Strain	Phy-Seq.	Isolated from	Geographic origin	Acc. #
GMI1000	I	Tomato	Guyana	GenBank: NC_003295, NC_003296
FQY_4	I	Soil	China	GenBank: CP004012, CP004013
Y45	I	Tobacco	China	GenBank: AFWL00000000
IPO1609	IIB-1	Potato	Netherlands	GenBank: CU914168, CU914166
UW551	IIB-1	Geranium	Kenya	GenBank: AAKL00000000
UW349	IIB-1	Potato	Brazil	GenBank: JQOI00000000.1
UW365	IIB-1	Potato	China	GenBank: JQSI00000000.1
UW491	IIB-1	Potato	Colombia	GenBank: JQSH00000000.1
RS2	IIB-1	Potato	N/D	EMBL: PRJEB8309
CFBP3858	IIB-1	Potato	Netherlands	EMBL: PRJEB8309
MolK2	IIB-3	Banana	Philippines	GenBank: CAHW01000040
CFBP1416	IIB-3	Plantain	Costa Rica	EMBL: PRJEB7434
CIP417	IIB-3	Banana	Philippines	EMBL: PRJEB7427
UW179	IIB-4	Banana	Colombia	EMBL: PRJEB7426
UW163	IIB-4	Plantain	Peru	EMBL: PRJEB7430
CFBP6783	IIB-4	Heliconia	French West Indies	EMBL: PRJEB7432
Po82	IIB-4	Potato	Mexico	GenBank: CP002819, CP002820
IBSBF1503	IIB-4	Cucumber	Brazil	EMBL: PRJEB7433
CFBP7014	IIB-59	Anthurium	Trinidad	EMBL: PRJEB8309
CFBP2957	IIA-36	Tomato	French West Indies	EMBL: FP885897, FP885907
K60^T^	IIA-7	Tomato	United States	EMBL: CAGT01000001
Grenada 9-1	IIA-6	Banana	Grenada	EMBL: PRJEB7428
UW181	IIA-6	Plantain	Venezuela	EMBL: PRJEB8309
B50	IIA-24	Banana	Brazil	EMBL: PRJEB7421
IBSBF1900	IIA-24	Banana	Brazil	EMBL: PRJEB8309
CMR15	III	Tomato	Cameroon	EMBL: FP885895, FP885896
PSI07	IV	Tomato	Indonesia	EMBL: FP885906, FP885891
BDB R229	IV	Banana	Indonesia	EMBL: FR854059 to FR854085
*R. syzygii* R24	IV	Clove	Indonesia	EMBL: FR854086 to FR854092

### Genomics

The complete and assembled genome sequence data used here are publicly available via the MicroScope web interface at www.genoscope.cns.fr/agc/microscope/home/. The Average Nucleotide Identity (ANIb) between genomes was calculated according to Konstantinidis and Tiedje [30], and the genomic distances were obtained after subtracting the ANIb values from 1. The Maximal Unique Matches index (MUMi) distances between genomes were calculated using the Perl script developed by Deloger et al. [29] using MUMmer genome alignment software [45]. The Genome-to-Genome Distance Calculator (GGDC) was used as previously described [31]. The DDH values were derived from the GGDC distances using formula 2 [31]. A phylogenetic network derived from the distance matrices produced with all three methods was created using SplitsTree4 software [46]. Distances matrices are available in Additional file 1.

### Anaerobic inorganic nitrogen metabolism assessments

Nitrate respiration and complete denitrification were assessed using slightly modified VDM medium [1, 47]. To decrease nitrate-independent anaerobic growth, we used casamino acids instead of yeast extract [22]. Additionally, we omitted nitrate from the base medium. This was done to allow assays to be conducted with and without nitrate under otherwise similar conditions. Where specified, 30 mM NO_3_^−^ (the concentration found in host plant xylem sap) was added in the form of filter sterilized KNO_3_ [22]. 1.5 mL of this modified VDM (+/− NO_3_^−^) was inoculated with a specified bacterial strain to a starting O.D._600_ of ~0.001 (~1 × 10^6^ CFU/mL). Tubes were incubated without agitation at 28 °C under anaerobic conditions in a BD GasPak anaerobic system. Seventy-two hours post inoculation, O.D._600_ measurements were taken from each culture. Two to four biological replicates were conducted per strain, per treatment (+/− NO_3_^−^). To determine if nitrate respiration contributed to anaerobic growth, O.D._600_ data were compared between + and − NO_3_^−^ treatments for each strain and depicted as a ratio. A ratio above 1 indicates that the strain grew better anaerobically when provided with NO_3_^−^. A ratio of 1 or below indicates that the addition of NO_3_^−^ did not enhance anaerobic growth, and that the strain did not respire with NO_3_^−^ under the conditions tested. Additionally, all cultures were visually assessed (± bubbles) over the course of 96 h for production of dinitrogen gas, the end product of complete denitrification [1, 48].

### Analysis of denitrification genes

Presence or absence of homologs involved in denitrification were determined in all sequenced strains (Table 1) using the MicroScope web interface and BLAST [49] to look for loci identified in the GMI1000 strain: *narG* (RSp0974); *nosZ* (RSp1368); *aniA* (RSp1503); and *norB* (RSp1505). Identity values were computed with the R package seqinr [50] after aligning amino-acid sequences with MUSCLE [51].

### Bacterial typing using matrix-assisted laser desorption ionization time-of-flight mass spectrometry

Seventy-three strains belonging to different phylotypes of the RSSC were characterized at the proteomic level using Matrix-Assisted Laser Desorption Ionization Time-Of-Flight Mass Spectrometry (MALDI-TOF MS). *Ralstonia eutropha* LMG 1199 was included in the analysis as an outgroup (Additional file 2). Bacterial strains were grown on Kelman broth supplemented with agar for 48 h at 28 °C. For whole-cell protein extraction, 1 μL of the bacterial biomass was collected and resuspended in a solution containing 50 % (*v/v*) acetonitrile (Acros Organics, Fair Lawn, NJ, USA) and 1 % (*v/v*) trifluoroacetic acid (Sigma-Aldrich, Saint Louis, MO, USA) in Milli-Q® ultrapure water (EMD Millipore Corporation, Billerica, MA, USA). The suspensions were vortexed twice for 10 s and centrifuged at 20,000 g for 10 min at RT. The supernatants were transferred and aliquoted into new tubes and stored at −20 °C until further analysis.

One microliter of the bacterial extracts was mixed with 1 μL of a saturated solution of α-cyano-4-hydroxycinnamic acid (Sigma-Aldrich), which was used as a matrix. The resulting sample/matrix mixture was deposited onto a stainless plate, dried at room temperature, and introduced into the MALDI-TOF MS instrument for analysis. The mass spectra profiles were obtained using a bench-top Microflex™ MALDI-TOF from Bruker Daltonics, including the Flex Control and Flex Analysis v3.3 software, at the Bacteriology Division of the CHU of St. Pierre, La Réunion. All spectra were obtained in linear positive-ion mode with an m/z range of 2000–20,000 Da. Each spectrum was calculated as the sum of 320 accumulated laser shots obtained after a spiral trajectory of the laser. For each sample, two bacterial extracts were obtained and measured in duplicate, and all the spectra were calibrated using a standard preparation of *Escherichia coli* DH5α, according to Bruker Daltonics.

All bacterial spectra were analyzed using FlexAnalysis software (Bruker Daltonics) to generate peak lists for each strain, and only peaks with a relative intensity greater than 2 % were considered for cluster analysis (Additional file 3). The peak lists were exported to a CSV file, exported to single files using a custom macro and loaded onto the SPECLUST web-service (http://co.bmc.lu.se/speclust/) to obtain a consensus peak list for all strains considered. For the consensus peaks, a peak match score (σ) width of ±3 Da was considered.

### Phylogenetic reconstruction using the MALDI-TOF data

The consensus peak list was formatted into a sequential Nexus binary file and loaded into MrBayes 3.2.2 software (http://mrbayes.sourceforge.net/). Phylogeny was obtained through Bayesian inference using the restriction data type (two states: absence or presence of a peptide denoted by a 0 or a 1, respectively), assuming that the frequencies of the two possible states had a Dirichlet (1.00, 1.00) prior. Bayesian analysis was performed in two runs using 8 Markov chains and 3,000,000 generations. The potential scale reduction factor implemented in MrBayes 3.2.2 was used as a convergence diagnostic. A majority-rule consensus tree (50 %) was obtained after discarding 25 % of the initial trees (burn-in = 0.25) generated before the stabilization of the log likelihood values of the data plotted against the number of generations. The trees were subsequently edited using FigTree v1.3.1 (http://tree.bio.ed.ac.uk/software/figtree/).

### Availability of supporting data

Genomes used in this study are available at the following repositories and accession numbers: GMI1000 [GenBank: NC_003295, NC_003296], FQY_4 [GenBank: CP004012, CP004013], Y45 [GenBank: AFWL00000000], IPO1609 [GenBank: CU914168, CU914166], UW551 [GenBank: AAKL00000000], UW349 [GenBank: JQOI00000000.1], UW365 [GenBank: JQSI00000000.1], UW491 [GenBank: JQSH00000000.1], RS2 [EMBL: PRJEB8309], CFBP3858 [EMBL: PRJEB8309], MolK2 [GenBank: CAHW01000040], CFBP1416 [EMBL: PRJEB7434], CIP417 [EMBL: PRJEB7427], UW179 [EMBL: PRJEB7426], UW163 [EMBL: PRJEB7430], CFBP6783 [EMBL: PRJEB7432], Po82 [GenBank: CP002819 CP002820], IBSBF1503 [EMBL: PRJEB7433], CFBP7014 [EMBL: PRJEB8309], CFBP2957 [EMBL: FP885897, FP885907], K60 [EMBL: CAGT01000001], Grenada 9-1 [EMBL: PRJEB7428], UW181 [EMBL: PRJEB8309], B50 [EMBL: PRJEB7421], IBSBF1900 [EMBL: PRJEB8309], CMR15 [EMBL: FP885895, FP885896], PSI07 [EMBL: FP885906, FP885891], BDB R229 [EMBL: FR854059 to FR854085], *R. syzygii* R24 [EMBL: FR854086 to FR854092].

## Additional files

Additional file 1:
**Genomic distance matrices.** Pairwise comparisons of 29 sequenced genomes from the *R. solanacearum* species complex using the ANI, MUMi and GGDC methods. The orange, blue and green cells represent strains clustered into species using the criteria specific to each method and corresponding to 70 % DDH. (XLSX 45 kb) Additional file 2:
**Bacterial strains used for proteomic analysis.** Phylotype and sequevar classifications for the strains in the *R. solanacearum* species complex were determined as previously described [14]. The sequenced strains are highlighted in green. (XLSX 47 kb) Additional file 3:
**MALDI-TOF peak lists.** Peak lists extracted from MALDI-TOF spectrum for 73 *R. solanacearum* strains with FlexAnalysis software (Bruker Daltonics). (ZIP 76 kb)

## Abbreviations

ANI: average nucleotide identity
GGDC: genome-to-genome distance calculator
BDB: blood disease bacterium
DDH: DNA–DNA hybridization
MALDI-TOF: matrix-assisted laser desorption/ionization-time of flight
MLST: multi locus sequence typing
MUM: maximum unique matches
RSSC: *Ralstonia solanacearum* species complex
